# Insufflation as an Auxiliary Method in Surgical Operations

**Published:** 1877-05

**Authors:** 


INSUFFLATION AS AN AUXILIARY METHOD IN SURGICAL OPERATIONS.—The *Revista Medico-Quirurgica*, No. 11, 1876, states that Dr. Moutes de Oca, clinical professor of surgery in Buenos Ayres, has devised a method by which operations are greatly facilitated in regions where Esmarch's bloodless method is impracticable, as, for instance, in the neck. The Professor has employed this method with the most brilliant results in the surgical wards of the *Hospital General de Hombres*, during the past two years, for the extirpation of tumors. The method consists in the insufflation or injection of air into the connective tissue surrounding the tumor, causing, the separation or isolation of the organs, and is performed in the following manner: A trocar is first introduced through the skin into the connective tissue in the vicinity of the tumor which is to be removed, the stylet is withdrawn, and the tube of a pneumatic pump connected with the canula, when two or three strokes of the piston will generally be found sufficient to cause the tumor to float on the distended cells. If the tumor be very large, it may be necessary to insufflate from two or more points. In performing the insufflation we should always take the precaution to compress the tissues at a certain distance from the tumor, in order to prevent the air from passing too far into the tissues. After this preparatory operation is completed, the surgeon cuts the integument covering the tumor with his bistoury, and then terminates the operation with his finger or with the handle of the instrument. "We have often had occasion to be present when the Professor has applied his method, and as often have we been obliged to admire its efficacy, and we cannot but feel amazed at the facility with which operations may be performed by this method. Among the operations that we have witnessed we remember some of great importance, that have proved, beyond doubt, the excellence of the method."—*Med. and Surg. Reporter*.

